# Facility staff perspectives on the implementation of Maternal and Perinatal Death Surveillance and Response in six health facilities in Kigoma, Tanzania

**DOI:** 10.1371/journal.pone.0349233

**Published:** 2026-06-01

**Authors:** Sarah Huber-Krum, Sarah Hartley, Patricia Spencer, Lauren Galioto, Abdulaziz Msuya, Florina Serbanescu

**Affiliations:** 1 Division of Reproductive Health, National Center for Chronic Disease Prevention and Health Promotion, Centers for Disease Control and Prevention, Atlanta, GeorgiaUnited States of America; 2 CDC Foundation, Atlanta, GeorgiaUnited States of America; 3 CDC Foundation, Tanzania; LMU München: Ludwig-Maximilians-Universitat Munchen, NEPAL

## Abstract

**Background:**

Maternal and Perinatal Death Surveillance and Response (MPDSR) is an important accountability mechanism for preventing avoidable deaths and addressing gaps in care. Health facilities in resource constrained settings often face barriers to implementing and sustaining MPDSR. The purpose of this study was to identify factors that may influence MPDSR implementation outcomes in health facilities in Tanzania.

**Methods:**

Semi-structured, in-depth interviews (IDIs) were conducted in January 2024 with 17 delivery care providers (e.g., doctors, midwives, anesthesiologists) and 5 health administrators who oversaw or facilitated the facility’s MPDSR process. The implementation outcomes framework and the Practical, Robust, Implementation and Sustainability Model (PRISM) were used to guide the study, the development of interview guides, and analysis. We analyzed transcripts using a multistage approach and the constant comparative method.

**Results:**

We identified several factors that may have impacted MPDSR implementation outcomes, which we grouped into three primary themes: (1) organizational and staff perspectives on MPDSR, (2) characteristics of the implementation setting, and (3) implementation and sustainability infrastructure. Subthemes included more specific barriers and facilitators that were related to MPDSR implementation outcomes. Prominent facilitators included positive perspectives of MPDSR, ongoing training and mentorship, and community engagement. Major barriers included lack of organizational readiness, resource, financial and other constraints, and blame culture.

**Conclusions:**

Identifying factors that influence MPDSR implementation outcomes is important for understanding barriers and facilitators to implementation. Fostering safe environments (i.e., no blaming), addressing barriers to staff participation and motivation, and implementing monitoring systems for MPDSR recommendations may help strengthen implementation outcomes and, ultimately, sustainability.

## Introduction

The majority of maternal deaths are concentrated in the African Region; in 2020, 202,000 of 287,000 global maternal deaths (70%) occurred in sub-Saharan Africa [1]. Tanzania has achieved substantial reductions in the maternal mortality ratio (MMR), from 760 in 2000–238 in 2020, which is lower than the regional average of 545 [1]. Main causes of maternal death in Tanzania include direct obstetric complications (84%) (e.g., obstetric hemorrhage, eclampsia, maternal sepsis), and indirect obstetric complications (16%) (e.g., anemia, cardiovascular disorders) that occur during pregnancy, childbirth, or within 42 days of delivery [2]. Monitoring maternal deaths, their causes, and the gaps in care that may have contributed to these deaths is critical to prevent future deaths.

Maternal Death Surveillance and Response (MDSR) is a system of continuous identification, notification, review, and analyses of maternal deaths to identify the medical and non-medical factors that contributed to a death and formulate “responses” to prevent future deaths [3]. A technical MDSR guidance was issued in 2013 by the World Health Organization (WHO) with the purpose of aiding global efforts to reduce maternal mortality [3]. Many countries, including Tanzania, have extended this surveillance system to include perinatal deaths (maternal and perinatal death surveillance and response or MPDSR) [3].

**Fig 1:**
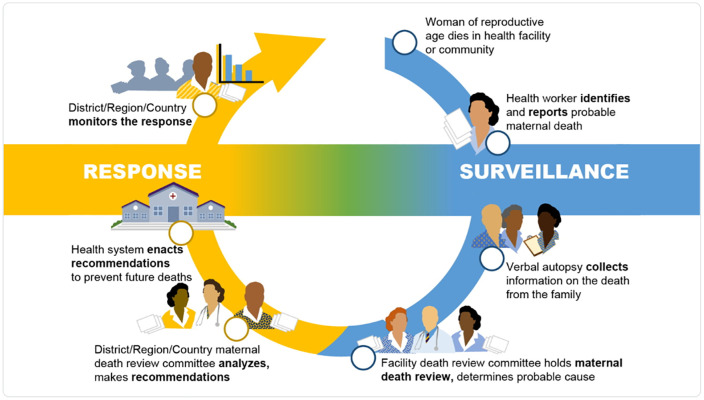
The Maternal and Perinatal Death Surveillance and Response (MPDSR) Cycle. Citation: Centers for Disease Control and Prevention. (2024). Global Reproductive Health: Maternal Death Surveillance and Response. https://www.cdc.gov/global-reproductive-health/php/maternal-death-surveillance-response/index.html.

The Tanzanian government has made concerted efforts and substantial investments to reduce maternal and perinatal deaths, partly through the implementation of MPDSR as an accountability mechanism for preventing avoidable deaths and addressing gaps in care [4]. Tanzania first developed maternal death review guidelines in 2006 and subsequently updated them to be aligned with the 2013 WHO guidance in 2015 and 2019. The national guidelines are geared toward eliminating preventable deaths in both facilities and communities and continuously documenting deaths in order to assess the magnitude, trends, and impact of mortality reduction strategies [5]. The guidelines require that a continuous cycle of surveillance, identification, notification, and review of maternal and perinatal deaths is used to collect information to identify and review deaths, develop action plans for prevention, and provide accurate data around maternal and perinatal deaths from facilities and communities (Fig 1). Action plans include recommendations stratified by identified problems and by the expected level of implementation. The facility MPDSR committee is expected to meet to identify, notify, and review all maternal and perinatal deaths within 7 days of occurrence and complete review forms that are sent to the district and regional health office together with the narrative case summaries, main gaps in care identified through the review process, and recommendations organized in action plans to address these gaps. The MPDSR committee meetings are multi-disciplinary and organized by the MPDSR focal point, chaired by the doctor in charge of the facility or the obstetric or neonatal ward, and documented by a secretary (e.g., the secretary may document attendance, adherence to code of conduct, feedback from previous recommendations, and cases to be reviewed). In addition, the meetings are often attended by the district and regional Reproductive Child Health Coordinators (RCHCo). While reviews are usually not attended by community members, the facility focal person and regional or district RCHCos are responsible for discussing with village health c‌‌ommittees any delays at the individual, household and community levels that may have contributed to maternal and perinatal deaths [5].

Similar to other low- and middle-income countries (LMICs), Tanzania faces barriers to implementing, scaling up, and sustaining components of MPDSR [6]. A 2018 study of narrative case summaries from death reviews highlights persistent challenges in the quality and utility of these reviews [5]. The finding that many summaries were incomplete and nearly half were classified as poor suggests that critical clinical and contextual information is often missing, potentially limiting the ability of review processes to accurately identify causes of death and inform effective responses [7]. Furthermore, the high proportion of non-specific action plans (54%) and the limited use of SMART recommendations (42%) indicate that even when issues are identified, they are not consistently translated into actionable and measurable steps [7]. Together, these findings point to systemic gaps in the implementation of maternal and perinatal death review processes, particularly in ensuring that reviews generate clear, high-quality, and actionable recommendations aligned with national guidance [5].

Three sub-national qualitative studies have identified several factors that adversely affect MPDSR implementation in Tanzania, including poor record-keeping, weak hospital systems, human resource constraints, ineffective implementation of action plans or non-development of action plans, delays in conducting reviews, and inadequate follow-up on recommendations [8–10]. At the same time, providers generally view MPDSR as acceptable and beneficial for addressing underlying causes of maternal deaths in facilities [8]. Additional facilitators of MPDSR implementation in Tanzanian facilities may include government prioritization of maternal health and availability of active MPDSR committees [10].

Existing research has largely focused on acceptability and fidelity, with limited attention to other key implementation outcomes, such as penetration and sustainability. This gap is important because even well-accepted maternal health interventions may fail to achieve population-level impact if they are not consistently implemented across facilities or sustained over time. Without understanding these dimensions, it is difficult to determine whether MPDSR processes are reaching all relevant facilities and maintaining functionality, which are critical for translating reviews into improved quality of care. Addressing this gap can provide actionable insights to strengthen health system performance by identifying where and why MPDSR implementation falters, thereby informing targeted strategies to improve coverage and continuity.

Our study was nested within a mixed-methods sustainability evaluation of a program that was implemented in Kigoma Region, Tanzania to reduce maternal deaths. We used qualitative in-depth interviews (IDIs) with facility staff to identify factors that may have affected MPDSR implementation outcomes. Identifying factors that influence the implementation of MPDSR in health facilities is important for understanding system level facilitators and barriers to full adoption of national guidelines.

### Parent study description and achievements

The “Reducing Maternal Deaths in Tanzania” Program (henceforth known as The Program) was a long-term public-private partnership carried out from 2006 to 2019 in Kigoma Region [11], which was one of the most underserved regions at that time [12]. The Program was funded by Bloomberg Philanthropies and Foundation H&B Agerup and implemented by the Government of Tanzania in collaboration with Thamini Uhai and EngenderHealth. The Program implemented a series of facility and community interventions to increase the availability of high-quality maternal and reproductive health services, including a substantial expansion of emergency obstetric and neonatal care (EmONC), and facility and community activities to improve and sustain access to maternal and reproductive health services and to create and sustain demand for maternal and reproductive health services [11,13]. A narrower focus of The Program was to improve the monitoring of maternal and perinatal deaths through MPDSR. In total, The Program trained approximately 30 clinicians representing the regional and district councils on MPDSR [13].

From 2013 to 2019, the US Centers for Disease Control and Prevention (CDC) partnered with The Program to conduct a comprehensive evaluation of the program activities and their impact on key maternal and perinatal health outcomes. The evaluation demonstrated a 43% reduction in maternal mortality between 2013 and 2018 and improvements in facility delivery care quality and clients’ satisfaction [13]. Additionally, the evaluation found a significant increase in the use of MPDSR review forms [13], suggesting improvements in the implementation of MPDSR in all facilities. In the final year of the Program’s implementation, a sustainability framework was introduced to transfer program management to the regional health authority, build capacity for providing continuous supportive supervision to providers and monitoring clinical care, and maintain components of The Program that were perceived as key in the maternal and perinatal death reductions, including MPDSR [11].

In 2022, the CDC Foundation, in partnership with CDC, assisted the Tanzania Ministry of Health (MoH) in conducting a sustainability evaluation of post-program services and outcomes in Kigoma region. The evaluation activities were modelled after the evaluation methods and approaches that were used to assess The Program impacts between 2013–2019. The aims of the sustainability evaluation were to 1) examine the status of processes and approaches that had been introduced by The Program during its implementation, and 2) examine the sustainability of obstetric care and maternal and newborn health benefits four years after the end of donor funding to The Program and its transition to the regional authorities.

A quantitative Health Facility Assessment (HFA) carried out in 2023 as a part of the sustainability evaluation documented the status of MPDSR implementation, fidelity, and sustainability in all health facilities [14]. Most, but not all, of the guidelines established by the national MPDSR guidance were implemented in Kigoma health facilities. Thus, a qualitative evaluation was carried out to provide additional context surrounding the factors that influenced MPDSR implementation outcomes.

## Methods

### Theoretical frameworks

We used two theoretical frameworks to guide this qualitative evaluation. First, to understand the factors impacting MPDSR implementation in health facilities in Kigoma Region, we applied the implementation outcomes framework [15]. The framework categorizes implementation outcomes into three distinct groups: implementation, service, and client outcomes. In this study, we focused on implementation outcomes, which are defined as the effects of deliberate and purposive actions to implement new practices that are necessary preconditions for attaining consequent desired changes in clinical outcomes [15]. Implementation outcomes include acceptability, adoption, appropriateness, costs, feasibility, fidelity, penetration, and sustainability (Table 1). For this analysis, we excluded costs. We applied these factors in the context of MPDSR implementation in health facilities located in a rural region. We used the framework to guide the development of study research questions, interview guides, and qualitative analysis.

**Table 1:** Definition of implementation outcomes as applied to maternal and perinatal death surveillance and response (MPDSR)^1^.

Implementation outcomes	Definition
Acceptability	The perception among implementation stakeholders that MPDSR is agreeable or satisfactory
Adoption	The intention or action to try or employ all recommendations for MPDSR as outlined in the United Republic of Tanzania Ministry of Health’s *Maternal and Perinatal Death Surveillance and Response Guidelines, 2019*^2^
Appropriateness	The perceived fit of MPDSR to address maternal and/or perinatal mortality in health facility settings
Feasibility	The extent to which MPDSR and MPDSR guidelines were successfully used or carried out within health facilities
Fidelity	The degree to which MPDSR guidelines were implemented as they were recommended in the Tanzania National Guidelines
Penetration	The integration of MPDSR within health facilities and their subsystems
Sustainability	The extent to which MPDSR is maintained or institutionalized within a health facility’s ongoing, stable operation

^1^Based on the implementation outcomes described in Proctor et al.’s (2011) taxonomy of outcomes in implementation research

^2^Citation: United Republic of Tanzania Ministry of Health (MoH). (2019). *Maternal and Perinatal Death Surveillance and Response Guidelines*. Dodoma, Tanzania: United Republic of Tanzania Ministry of Health

**Fig 2:**
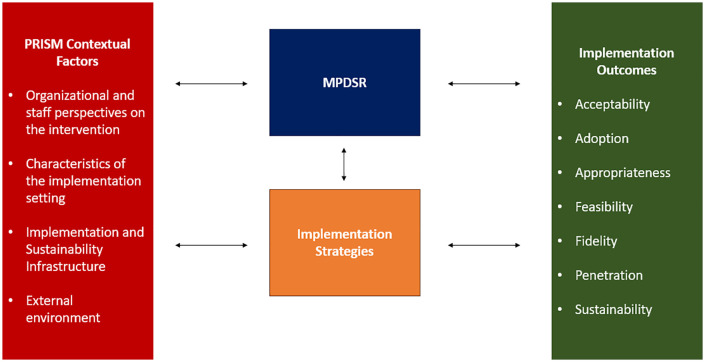
Depiction of how the Practical, Robust Implementation and Sustainability Model (PRISM) and the Implementation Outcomes Framework address maternal and perinatal death surveillance and response (MPDSR) sustainability. Citations: Proctor, E., Silmere, H., Raghavan, R., Hovmand, P., Aarons, G., Bunger, A., Griffey, R., & Hensley, M. (2011). Outcomes for implementation research: conceptual distinctions, measurement challenges, and research agenda. *Adm Policy Ment Health*, *38*(2), 65−76. https://doi.org/10.1007/s10488-010-0319-7; Feldstein, A. C., & Glasgow, R. E. (2008). A practical, robust implementation and sustainability model (PRISM) for integrating research findings into practice. *Jt Comm J Qual Patient Saf*, *34*(4), 228−243. https://doi.org/10.1016/s1553-7250(08)34030-6.

We also used the Practical, Robust Implementation and Sustainability Model (PRISM) to guide the qualitative analysis and presentation of the results. PRISM is an implementation science framework that can be used to understand and address the contextual factors that affect a program (such as MPDSR implementation), as well as strategies to enhance the reach, effectiveness, adoption, implementation, and maintenance of programs [16]. There are four contextual PRISM domains, including (1) organizational and staff perspectives on the intervention, (2) the characteristics of the implementation setting, (3) implementation and sustainability infrastructure, and (4) the external environment (Fig 2). In this analysis, we focused on the first three context domains, because we did not obtain the perspectives of community members or probe about external factors such as market forces, economic factors or the policy and legal environment.

### Study setting

**Fig 3:**
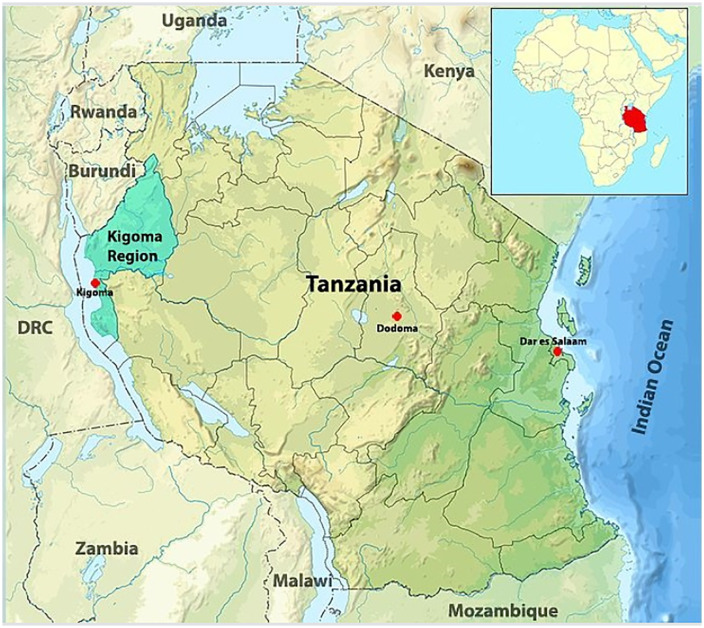
Map of Tanzania and location of Kigoma Region. Citation: CDC. (2024b). *Global Reproductive Health: Maternal and Reproductive Health in Tanzania Program*. https://www.cdc.gov/global-reproductive-health/php/tanzania-program/index.html.

Kigoma Region is located in northwest Tanzania (Fig 3). The region has a low population density and is predominately rural (82% of the area is designated as rural) [17]. Transportation and public infrastructure present an ongoing challenge across all districts, with Kigoma Rural district having the least access to health services due to fewer health facilities and passable roads.

Kigoma Region has made substantial progress in improving access to maternal health services, despite its remoteness and infrastructure issues. According to the 2022 Tanzanian Demographic and Health Survey (TDHS), about 94% of live births were reportedly delivered in a health facility, substantially higher than neighboring regions (for example, Katavi at 67%), and 96% were delivered by a skilled provider [18]. Additionally, almost 80% of women received any antenatal care by a skilled provider, and 57% received a postnatal checkup within 42 days after birth [18]. Despite this progress in maternal health care, indicators of sexual and health services have lagged. Approximately 23% of reproductive aged women used modern contraception, and the total fertility rate (TFR) was 5.8 children per woman, one of the highest for the country [18]. The mean number of children ever born to women aged 40–49 was 7.1, which was the third highest in Tanzania [18].

### Study design and data collection procedures

Qualitative, semi-structured in-depth interviews (IDIs) were conducted to understand how facilities conduct MPDSR and the barriers and facilitators to implementation outcomes. Six hospitals located in Kigoma Region, including three government hospitals, the largest private hospital, the public health center with the highest volume of deliveries, and the private health center with the highest volume of maternal deaths, were purposively selected for participation. All facilities had active MPDSR committees consisting of obstetricians, medical doctors, clinicians, nurses, midwives, managers, and other supporting staff. Facilities were selected based on the likelihood that they would have multiple maternal and perinatal deaths to review during the data collection period.

Two to 3 participants were purposefully selected from each facility based on their involvement with their facility’s MPDSR committee (e.g., MPDSR focal person, MPDSR chairperson, and MPDSR secretary). Additionally, 5 health administrators responsible with the district and regional coordination of the MPDSR system were interviewed. The sample size was estimated based on what would be sufficient to achieve saturation in themes and study aims [19]. In total, 17 delivery care providers (e.g., doctors, midwives, anesthesiologists) and 5 health administrators who oversaw or facilitated the facility’s MPDSR process were interviewed in January 20–31, 2024 in English or Swahili. Participants were interviewed in Swahili if they did not speak English. All participants who were invited to participate in the study were interviewed. Interviewers were hired and trained on the study procedures, qualitative interviewing, ethical considerations, informed consent process, and interview guiding questions. Interviewers were Tanzanian physicians from Dar es Salaam with no prior relationships to study participants. Interviewers contacted hospital administration by phone to inform them about the interviews, briefly explained the purpose of the interviews, and requested and obtained participation consent in writing.

The Tanzania National Guidelines for MPDSR [5] and the theoretical frameworks were used to develop a comprehensive interview guide in English. Tanzanian colleagues and collaborators reviewed and provided feedback on the guide. The guide was translated from English to Swahili by Tanzanian partners who were fluent in both English and Swahili. Then, the guide was back-translated to confirm that the meaning of questions was preserved. The interview guide covered topics related to MPDSR implementation, action plan formulation, and barriers and facilitators to MPDSR.

### Ethics approval and consent to participate

Before each interview, participants were asked to provide verbal consent to take part in the study. Consent was audio recorded. Interviews were conducted in private locations at the facilities, with no incentives. Interviewers made notes during the interviews that were used during transcription/translation to add contextual details. Interviews were audio recorded with participants’ permission and subsequently transcribed and translated to English (if applicable) for analysis. Interviews lasted between 30–60 minutes.

This project was approved by the National Institute for Medical Research (NIMR) in Tanzania as one activity among a larger set of activities for the sustainability evaluation of the *Program to Reduce Maternal Deaths in Tanzania* (Ref. NIMR/HQ/R.8a/Vol. IX/1039). This activity was reviewed by CDC and was conducted consistent with applicable federal law and CDC policy (per 45 C.F.R. part 46; 21 C.F.R. part 56; 42 U.S.C. Sect. 241(d), 5 U.S.C. Sect. 552a, 44 U.S.C. Sect. 3501 et seq).

### Analytical strategy

We used NVivo 14 to manage, code, and interpret the data using thematic content analysis [19]. We applied a multistage analytic approach to index and code the data. In the first stage, we reviewed transcripts, interviewer notes, and audit trails (e.g., field notes, interview tracking forms, debriefs, protocols) and summarized the content according to broad themes guided by the theoretical frameworks. We prepared an initial list of parent codes and definitions based on the study research objectives, contents of the interview guides, the existing literature on MPDSR implementation, the implementation outcomes framework, and PRISM domains. We also developed a list of inductive codes derived from the review and pilot coding of a subset of transcripts. Four coders reviewed the initial codebook in detail and resolved any issues/discrepancies in codes and definitions. All study authors met to provide collaboration feedback on the codes and definitions. A final codebook was developed when all coders and researchers involved in the project agreed upon each code and the definition for each code.

In the second stage, we used the constant comparative method to apply codes to all text [19]. We used analytical memos to summarize case details and to highlight particularly rich narratives and emergent themes. Coders met regularly to review coding decisions and come to an agreement on major themes to ensure analytical rigor and consistency. Coders also discussed any coding issues during regular meetings and resolved discrepancies.

As a last step, we organized the data into thematic categories, preserving the context of the original text. Specifically, we sorted verbatim quotes into categories that described domains and subdomains of the research questions and the theoretical frameworks. For example, we organized quotes related to sustainability into a “sustainability theme” and quotes related to organizational and staff perspectives into a “organizational and staff perspective” theme. Researchers met regularly to discuss major themes and subthemes and come to a consensus. Then, we organized each into a comprehensive outline with descriptive quotes. We present quotes verbatim wherever possible. If changes were made to verbatim quotes, we made notations and provided additional contextual information if it did not jeopardize a participant’s anonymity. We organized results by three major themes that align with the PRISM framework, including (1) organizational and staff perspectives on the intervention, (2) characteristics of the implementation setting, and (3) implementation and sustainability infrastructure. Within each major theme, we present a narrative of subthemes and describe relevant connections to implementation outcomes. We include an appendix with additional quotes stratified by subtheme (S1 Table).

## Results

### Participant demographics

Many participants were Doctors of Medicine in their current facility (40.9%; Table 2). Additionally, 22.7% were Nursing Officers (nurses who have a bachelor’s degree in nursing), 31.8% were Assistant Nursing Officers (nurses who have advanced diplomas or diplomas in nursing), and 4.5% were Enrolled Nurses (nurses who have certificates in nursing). Over half of participants (54.5%) had been in their current position in their facility for 1–3 years. Most participants received formal training on MPDSR (63.6%).

**Table 2:** Participant Demographics (n = 22).

Characteristic	n	%
Professional position at facility		
Doctor of medicine	9	40.9
Nursing officer^1^	5	22.7
Assistant nursing officer^2^	7	31.8
Enrolled nurse^3^	1	4.5
Years in position		
<1	5	9.1
1-3	12	54.5
4+	5	9.1
Received formal training on MPDSR		
Yes	14	63.6
No	8	36.4

Abbreviations: MPDSR = maternal and perinatal death surveillance and response

^1^Nursing officers have bachelor’s degrees in nursing

^2^Assistant nursing officers have advanced diplomas or diplomas in nursing

^3^Enrolled nurses have certificates in nursing

### Major themes

#### Organizational and staff perspectives on the intervention.

According to PRISM, organizational and staff perspectives on the intervention may include characteristics such as the intervention’s perceived evidence strength, compatibility with existing workflow, and perceived organizational readiness for the program [16]. It may also include recipients’ perspectives (in the case of MPDSR, those who participate in reviews), such as the perceived estimated impact and ease of use [16]. We identified three subthemes, including organizational and staff perspectives on MPDSR’s benefits and impact, organizational and staff perspectives on MPDSR’s ease of use, and organizational and staff perspectives on organizational readiness to implement MPDSR.

### Organizational and staff perspectives on MPDSR’s benefits and impact

Participants were highly enthusiastic about MPDSR and its perceived benefits, which included reduction of maternal mortality through the identification of the direct and indirect causes of death and addressing those causes through recommended action plans. Participants also noted that MPDSR addressed identified gaps in the healthcare system, improving providers’ technical skills and knowledge, and, ultimately, improving quality of care. Participants perceived that acceptability of MPDSR positively influenced adoption and sustainability. A nursing officer with 2 years of experience as a district reproductive and child health coordinator commented on the utility of MPDSR:

“[MPDSR] has helped to equip people, i.e., he wasn’t aware of something, and you then enlighten him, then through that you equipped him. And also, it improves service delivery at the facilities. For example, perhaps in MPDSR we have identified that mothers in certain facilities are not sent for urine, stool, or hemoglobin investigations; it is just malaria rapid diagnostic test (MRDT) and blood test for syphilis that are examined. So, through MPDSR we tell our facilities that [more] investigations should be present in your facilities hence because of that we improve service provision in facilities.”

### Organizational and staff perspectives on MPDSR’s ease of use

Participants described weaknesses in MPDSR processes and systems that made use cumbersome. Some participants noted that at times committee members fail to report back on recommendation implementation, despite national guidelines requiring committee members to report back on progress. Participants noted that this could be because the person responsible for implementation would fail to appear and provide a report on progress or because they did not implement recommendations in the given time frame. However, participants noted it was not entirely possible to know why reporting back does not happen, because no action plan tracking or monitoring systems are in place. A nursing officer who serves as their facility’s MPDSR secretary noted:

“They give feedback to some extent. However, we are still lagging. We are not that good in giving action plan feedback. That is what I can say. In a such a way that sometimes they give feedback, you might see evidence of attending training, there are photos which were taken... And for example, this date we did training in a certain department or health education was provided, so there is a plan of preparing a topic.”

A common logistical challenge that participants reported was the involvement of multiple facilities in a death review meeting. Per the Tanzanian MPDSR guidelines [5], all facilities involved in the care of the deceased must be present at a review. Participants reported that it is difficult to collect the details of the death that are needed for the review process from another facility and to involve staff from other facilities in a review. Participants noted this was mainly due to resource and financial constraints, such as needing to provide transportation or a per diem for staff from other facilities. A medical doctor and MPDSR focal person commented on the challenges they face in tracking action plans and in convening meetings when multiple facilities are involved in a case:

“Let’s say we don’t own [an action plan], because a patient might have started their care at facility X and some of the action plans should be implemented at that facility X. So, we do not own the full action plan as a facility. So, to follow its implementation, we face difficulties. And, during the review process, we might need staff from that facility and, sometimes, they do not come to our review meetings... And another challenge is because we are a referral hospital, so most of our cases started somewhere else. So, we receive them at late and complicated stage. So, in some circumstances, we need the representative from the primary center where the patient started receiving care. So, in most of the reviews, it is difficult to get those people from the other facility to get the information.”

### Organizational and staff perspectives on MPDSR organizational readiness

Participants’ perspectives on organizational readiness to conduct MPDSR reviews and implement action plans and recommendations varied. Most participants described MPDSR meetings as well attended and collaborative. They reported that through the death review process, action plans are formulated with input from most committee members. This collaborative and participatory nature of the MPDSR meetings was perceived to strengthen action plans and recommendations, which are a part of MPDSR response. A medical doctor who served as a MPDSR chairman said:

“The action plan is prepared by the entire committee... Then, we all come with recommendations on what to do, what was the problem and the solution of the problem, and the responsible person and timeframe. It is highly participatory. We allow every member to contribute. It is an interactive process.”

On the other hand, lack of readiness to implement recommendations was recognized as a barrier. Participants reported several potential reasons for lack of readiness. For example, some participants reported that not all facility staff might understand the purpose of MPDSR, and thus potentially be unwilling to implement recommendations. Other participants highlighted that lack of knowledge about MPDSR can result in staff feeling uncomfortable about participation in meetings. A medical doctor and MPDSR focal person reflected on the potential reasons for nonattendance at review meetings:

“Most staff don’t really know the review process, and sometimes some of the [staff] might be uncomfortable attending. They may think that it is a judgment or what. So, some of the people don’t understand really the aim of MPDSR, and the team is always changing. There are new people joining the system. So, I think for those who are working in this obstetrics and gynecology department, especially and pediatric department especially neonatal ward, should have at least quarterly, quarterly briefing of what is the aim of MPDSR, so that everybody knows its aim is to identify just the gaps. It’s not for blaming people.”

Participants also reported that some facility staff were not fully committed to MPDSR and/or to implementing action plan recommendations, highlighting potential issues with fidelity and adoption. This lack of commitment and/or understanding may affect the MPDSR process. For example, staff who lack commitment to MPDSR might forgo attending meetings or not support collecting the information needed, therefore, hampering data collection efforts for the death review. A medical doctor and MPDSR chairperson noted:

“The motivation for the members [is a challenge in conducting reviews], because sometimes it may happen that we have few members who attend because they need some motivation at that time. Though we are trying whenever we have money to pay them to motivate them to attend. So, if one day you miss the motivation, next time you will have few members. Yeah, that’s our people.”

### Characteristics of the implementation setting

Characteristics of the implementation setting include any factors that affect a facility’s ability to successfully implement an intervention; these may include support and communication, and characteristics of the intervention and of participants that impact implementation outcomes (Feldstein & Glasgow, 2008). In our application of PRISM to facility based MPDSR, we identified two characteristics that affected MPDSR implementation outcomes, including (1) limited human, financial, and other resources and (2) competing roles and responsibilities of staff.

### Characteristics of the implementation setting: limited human, financial, other resources

Limited resources affected a variety of MPDSR processes, including death review, implementation of recommendations, and monitoring progress of implementation. Some participants reported that staff/facilities are not always provided resources and/or support to implement certain recommendations, and this could lead to committees only proposing achievable recommendations. A nursing officer with 10 years of experience in their current role and who served as a MPDSR coordinator reflected on how their facility was developing action plans that they know they have the resources to implement:

“When we make action plans, we consider those which are feasible; they can be completed. Because there are some gaps which are above our capability. So, when we make action plans, we consider those which we are capable [and have the resources] to complete them…Yes, we have failed to follow up action plans due to lack of resources.”

Financial constraints were also a commonly mentioned barrier to MPDSR implementation, often impacting a committee’s ability to hold meetings, to make SMART recommendations, and to follow up on action plans. Participants reported clear constraints for holding meetings; for example, some facilities could not provide travel funds for meetings (which limited participation) or refreshments during meetings (which are often long and intensive), disincentivizing committee member attendance. Additionally, several participants reported that committees will select and implement recommendations with the least costs. A medical doctor who served as a MPDSR chairperson said:

“Sometimes [we have problems implementing action plans], because it depends on the resources needed on implementing it; if it’s more cost efficient. For example, we have one generator before I arrived here. It happened that a patient was in the theatre, then the electricity went off and the generator was not working. You see in this instance, the action to be taken is to buy another generator, which is of course very costly. So, you find that you can’t even solve the problem because of the cost to solve it… If the resource is costly, then it is difficult to solve it. However, if it is less costly, then we can easily solve it. So, in short it is economic issues like that…We can make it as a long-term goal [to save money], but while the services are continuing, the same problem will be identified again.”

### Characteristics of the implementation setting: competing roles and responsibilities

Competing roles and responsibilities of staff impacted their ability to implement MPDSR processes (for example, holding or participating in meetings and/or gathering information for death reviews). Staff described numerous constraints on their time, which led to them juggling multiple priorities, tasks, and responsibilities. In addition, participants described how time constraints impacted their ability to implement recommendations and monitor implementation of action plans. Time constraints were also exacerbated by shortages of human resources in the region. A medical doctor who served as a medical officer in charge and MPDSR chairperson reflected on these tensions:

“One of the challenges [in implementing MPDSR] is time. As you know, I am the chairperson and I’m the medical officer in charge, and I have a lot of things that are my responsibility. So, some time you can even fail to be at work directly because even some of the meetings I can send the one who is left there in my office is acting medical officer in charge to attend as the chairperson. However, then I can’t know directly on the action plan where they previously ended as the chairperson, so that’s a challenge to me.”

Full staff participation in MPDSR processes, such as attending reviews or providing information to committees, was also reported as a barrier to adoption and sustainability. Participants reported several reasons for not attending reviews such as struggling to meet competing priorities and the timeline of death reviews that were not always compatible with staff schedules. A nursing officer and MPDSR secretary explained:

“Sometimes you might find that the one who was assigned to the action plan is absent at that moment, and this is a challenge to following up action plans. Therefore, it becomes difficult to get feedback on the progress of the action plan. Another issue is getting committee members to attend meetings. It is very challenging because they have been occupied with a lot of responsibilities, so to get them it become a challenge.”

### Implementation and sustainability infrastructure

Implementation and sustainability infrastructure are factors related to ongoing support, such as training, dedicated implementation teams, ongoing audit and feedback, and dedicated resources and plans for sustainability (Feldstein & Glasgow, 2008). We identified six factors related to infrastructure that acted either as barriers or facilitators to MPDSR implementation and sustainability. They include (1) training and mentorship, (2) facility leadership in organizing, chairing, and supporting multidisciplinary meetings that follow rules of conduct, (3) data analysis and dissemination, (4) designing quality improvement activities and budgeting for their implementation and follow up, (5) district and regional support, and (6) community engagement.

### Implementation and Sustainability Infrastructure: training and mentorship

Participants reported that initial and ongoing training helped staff understand the purpose and goals of MPDSR and better understand its processes. Some participants also mentioned that mentorship of staff with limited experience with MPDSR helped them better understand why it is needed and corrected misinformation and/or fear about the process. Trainings also supported fidelity. Participants reported that after training, more staff understood the importance of confidentiality and anonymity. Many participants reported that there were ongoing training needs in their facility that were not currently being met. A nursing officer and a reproductive and child health coordinator explained:

“Before we got MPDSR training, as I had said earlier, we were taking disciplinary actions like we write them warning letters or we deduct the number of off duties. When he causes a fresh still birth, we deduct a day off and he gets back to work. But after training, it helped us to understand how to conduct MPDSR reviews and follow-up on the actions and if they are implemented on time.”

### Implementation and Sustainability Infrastructure: facility leadership in organizing, chairing and supporting multidisciplinary meetings that follow rules of conduct

Each MPDSR committee has multidisciplinary meetings that are organized and chaired with support from the facility, including the provision of an adequate and private space for the meetings and mobilizing the committee members to attend though WhatsApp messages. In some facilities, leadership is less supportive of MPDSR, meetings are conducted with few members present and with varying degree of documentation and participation. A medical doctor who served as a MPDSR chairperson and focal person said:

“Some authorities may question the importance of the review. Not all, but some may question the importance of the review. Some ask, “does it help anything?” We’re doing all these meetings, but we still see deaths occurring. Is it helpful?” Some authorities I know I’ve been cornered by several officers and they’re telling me you’re wasting your time because deaths are still occurring…some of them just don’t know, the meaning, MPDSR.”

In addition, many participants reported that their facility tried to maintain the principles of “No Name, No Blame and No Shame,” which is considered essential for sustained MPDSR implementation (WHO, 2021). Additionally, many participants reported that committees implement strategies to create an environment conducive to fostering confidentiality and anonymity. For example, some participants reported that at the start of a meeting, they will review MPDSR principles aloud. Other participants reported instances in which they had to support colleagues in maintaining these principles by calling out deviations during meetings or having direct conversations with other members about blaming and shaming. Participants also largely reported that a code of conduct is shared with all committee members prior to meetings. A nursing officer and MPDSR coordinator said:

“The environment of meetings should be calm, and the meeting should involve only the invited members so as to maintain privacy. Before the meeting we explain that we will discuss details about a death, but we emphasize privacy. What we do is to emphasize and discourage on linking the information of the one who was involved in the caring of the client… We usually emphasize that, before we start our meeting, that someone should not feel shame or feel bad in the meeting, because our sole purpose is to fix the problem.”

However, some participants reported that shaming and blaming may still occur during or after meetings. Some participants also provided examples of disciplinary actions taken by facility leadership against staff after MPDSR meetings. Disciplinary actions seemed to range in severity, from subtle (e.g., letters or one-on-ones) to more extreme (e.g., temporary job reassignment). A nursing officer who served as a MPDSR focal person reported:

“When we give punishments, they become alert with a vivid example that they saw someone had been punished. Most of [the punishments] are job rotation. For example, if I mess up here, I am shifted to mortuary, so when you are sent to mortuary it is an adequate punishment. You are told to do cleanliness and so and so, which is a real torture. For example, you are a nursing officer with your good education, you are sent to reception that every morning you sweep the compound; it is an adequate punishment… The main reason [for punishments] is causing harm to patients or even causing death due to negligence, so we don’t tolerate on that.”

### Implementation and Sustainability Infrastructure: data analysis and dissemination

Many participants reported that their facilities used data collected as a part of the MPDSR process to monitor trends in maternal death and identify and track causes of death. Participants also noted that they used these data to identify key gaps in their facilities and/or communities to address as part of action plans. Many participants reported that they shared data with facility staff through postings or reports. However, fewer participants reported that they shared information related to maternal deaths with surrounding communities. If facilities did engage in community dissemination, they often provided this information through community leaders. Among participants who reported that their facilities did not engage in data analysis or dissemination, time and human resource constraints were the most cited barriers. A nursing officer who had been in her current position 2 years and served as a reproductive and child health district coordinator reported:

“We analyze and create reports [of the data collected from the reviews]. For example, in this quarter, I perhaps got two perinatal deaths from prematurity complications and from birth defects. Previously, we had like 5 deaths in a quarter. [We displayed the data on maternal and perinatal mortality] for staff. Also, if a death has occurred, staff will read about it in the morning report, and they will know.”

### Implementation and Sustainability Infrastructure: designing quality improvement activities and budgeting for implementation and follow up

Most participants reported that their facilities have quality improvement teams and that these teams follow up on action plans and recommendations. Some participants discussed how they budget for resources that need long term solutions through annual health facility plans. Quality improvement teams will follow up on action plan implementation but not necessarily all the time. Quality improvement team members may also be on MPDSR committees. A nursing officer who served as a maternal and child health services coordinator said:

“You might find that there are some of the challenges in the district and we failed to solve them then we insist to add them in their district annual budget, then we insist them to add on facility annual budget, so as they could solve them. For example, we have challenges of anesthetic machine, we advised the district level to add them in their annual plan. While for us we tried to find donation from stakeholders, but if the failed to achieve then in region level we will include it in our annual budget plan.”

### Implementation and Sustainability Infrastructure: district and regional support

Most participants reported that the district or region officials/coordinators follows up on action plans. However, many participants could not detail the process by which this follow up occurs, despite that district and regional coordinators are required to attend MPDSR meetings. Many reported that the district/region follows up via a phone or an in person visit in which they ask if certain recommendations were implemented or not. It was unclear whether the district/region reviews all action plans or the frequencies of follow ups (e.g., every quarter or every month). A medical doctor who served as a MPDSR focal person said:

“The district, especially the district RCHCo, makes a quarterly follow up on the implementation of action plans. So, they assess whether the action plans specified for all deaths that occurred within that quarter were they implemented or not… I think then the DRCHCo communicates with the region.”

### Implementation and Sustainability Infrastructure: community engagement

Community engagement was another factor that may influence implementation outcomes. Overall, participants reported that they did provide information about recommendations to those living in surrounding communities, but typically only if a recommendation was related to external factors, such as lack of knowledge about maternal health issues in the community, or if a maternal death occurred because of an external or community related factor (e.g., delaying care). While some participants reported that they did provide communities with information, reports of in-depth engagement were limited. Most participants reported that they do not share data or reports with community members about trends in maternal death or its causes and/or develop dissemination products for local communities, specifically. This was despite that many participants recognized the importance of educating communities about maternal death, addressing delays in seeking care, and addressing community beliefs and norms that impact maternal mortality. A medical doctor who served as a MPDSR chairperson and focal person reflected on the challenges of community engagement:

“Most of time, the challenges to implementing response are beyond our control at the facility. The biggest challenge is the community. Most community members are not aware of antenatal care, perinatal care, and postpartum care. So sometimes, they stay at home, or they come too late. So, to assist them is very difficult. And for us, the capability is to go to the community to assist them is difficult. Even though we have our community health workers that follow up patients, but they are not enough.”

## Discussion

MPDSR is an approach to identify, examine, and create actions/recommendation to prevent maternal deaths. Several studies have explored the multilevel factors needed to sustain practice of MPDSR [20]. Using the implementation outcomes framework and PRISM, we identified factors that may have affected MPDSR implementation in rural Tanzania. Prominent facilitators included positive perspectives of MPDSR, ongoing training and mentorship, and community engagement. Major barriers included lack of organizational readiness, resource, financial and other constraints, and blame culture.

We found that providers were supportive of MPDSR and perceived it as a beneficial strategy to prevent maternal death and improve the quality of care. Additionally, the collaborative nature of MPDSR enhanced action plans and recommendations. A qualitative, sub-national study also found that Tanzanian providers were supportive of MPDSR in facility settings [8]. In 2023, 93% of health centers and 91% of hospitals in Kigoma Region reported that they have a formal system for reviewing maternal deaths [14]. This widespread adoption of MPDSR may be partly attributable to high levels of acceptability. Acceptability of health interventions is a key consideration in implementation and sustainability [21]. If MPDSR is considered acceptable, then facilities and providers may be more likely to adopt national guidelines and continue to support the death review process over the long-term [6,21]. Positive perceptions of the consequences of MPDSR (e.g., improving health systems and quality of care) are also important to MPDSR implementation and sustainability [20].

Fidelity to the principles of “No Name, No Blame and No Shame” and confidentiality is widely recognized as essential to MPDSR implementation and sustainability [6,20,22]. In this study, participants described how breakdowns in these principles, particularly the persistence of blame culture, directly undermined multiple implementation outcomes. Specifically, when confidentiality is not fully maintained, providers may fear blame, reprimand, or professional consequences, which discourages open discussion of clinical decision-making and limits the completeness and accuracy of case reviews. This, in turn, reduces the quality of recommendations generated and weakens follow-through, thereby affecting both fidelity and sustainability. Participants also highlighted how structural constraints, such as limited human resources and hierarchal workplace dynamics, contribute to these challenges. Hierarchal relationships may also inhibit lower-cadre staff from speaking openly, further constraining honest dialogue and shared accountability. These dynamics create conditions in which responsibility may be avoided or shifted, ultimately limiting the effectiveness of MPDSR processes.

Human resource constraints were also described as a cross-cutting barrier affecting not only meeting dynamics but also the broader MPDSR cycle, including the development, implementation, and monitoring of recommendations. Staffing shortages limited the ability of committees to convene regularly, reduced participation in meetings, and constrained follow-up on action plans. These challenges were particularly pronounced for perinatal death reviews, which occur more frequently than maternal deaths, making it difficult to adhere to national guidelines recommending review within seven days [14,23].

Consistent with prior studies in Tanzania [8,20,22], our findings suggest that resource constraints and lack of anonymity reinforce blame culture; however, this study further demonstrates how these factors operate in practice to disrupt key implementation processes, including participation, quality of review discussions, and actionability of recomme‌‌ndations. Addressing these barriers, will require not only reinforcing the principles of confidentiality and blame-free review through training, mentoring, and supportive supervision [6,24], but also strengthening human resource capacity and institutional structures to enable consistent, high-quality implementation. Investments in staffing, supervision, and systems to timely and regular reviews may help foster a culture of trust and improve the translation of MPDSR findings into meaningful improvements in care.

WHO’s MPDSR technical guidance defines the primary purpose of MPDSR meetings to identify and address gaps in care through the formulation and implementation of SMART recommendations [22]. We identified several factors that affected formulation, implementation and monitoring of recommendations under several subthemes (e.g., “*Organizational and staff perspectives on MPDSR’s ease of use”, “Organizational and staff perspectives on MPDSR organizational readiness”, and “Characteristics of the implementation setting: limited human, financial, other resources”)*. Resource and time constraints may result in incomplete recommendations or prioritizing those that are the easiest to implement. Participants mentioned that facilities often lack the resources needed to implement and monitor progress in completing recommended actions. Participants also reported that staff motivation affected recommendation monitoring and may result in avoidance or failure to report back on implementation progress. Other qualitative, sub-national studies conducted in Tanzania found that health facilities may not follow national guidelines on the development, implementation, and monitoring of recommendations [7,9,10]. Lack of (or inadequate) monitoring systems for MPDSR action/recommendations remains a critical issue in LMICs [25,26]. WHO recommends that monitoring systems for MPDSR include tracking whether proposed recommendations have been implemented, whether implementation was timely, whether the recommendations have achieved the desired results, and what problems were encountered if the desired results were not achieved [22]. Studies of MPDSR implementation similarly report that partner engagement in recommendation monitoring is needed [20]. Monitoring and reporting back on recommendation progress may also serve to motivate MPDSR committee members to sustain MPDSR reviews, take ownership and responsibility for implementation, and make recommendations that are actionable within available budgets and/or identify resources for implementation [6,20].

While this study is grounded in the Tanzanian context, our findings are broadly consistent with evidence from other low- and middle-income countries, where MPDSR implementation has similarly been shaped by health system constraints, including workforce shortages, resource limitations, and challenges in fostering blame-free review environments [6,20,26]. Studies from sub-Saharan Africa and other LMIC settings have likewise identified the importance of supportive supervision, strong leadership, and functional monitoring systems in translating MPDSR processes into improved quality of care and outcomes [6,20,25,26]. At the same time, the specific ways in which these factors manifest are highly context dependent. Tanzania’s long-standing national commitment to MPDSR and relatively high levels of facility-based adoption provide a distinct implementation environment, underscoring the importance of context-specific analyses, such as this study, to understand how global guidance is operationalized in practice.

Due to the sampling design and qualitative nature of the study, our findings can only be generalized to the parent population from which the sample was drawn. Thus, findings cannot be generalized to other health facilities beyond the six included in this study. Additionally, findings are limited to MPDSR that occurs at health facilities, and we cannot generalize to community MPDSR. We also did not obtain the perspectives of community members or probe about external factors such as market forces, economic factors or the policy and legal environment. Thus, our findings were limited to internal organizational and staff perspectives. Additionally, we did not collect detailed information on the timing or recency of MPDSR-related training among participants, which may influence how training is retained and applied in practice. Lastly, respondent views may not reflect those of other staff at the health facilities who were not included in the study and may also be subject to social desirability bias.

This study did not explicitly examine implementation strategies (e.g., specific interventions used to support MPDSR implementation such as training modalities, supervision models, or incentive structures). While implementation strategies are critical determinants of implementation outcomes, our study was designed to explore perceived barriers and facilitators across implementation domains rather than to systematically assess or compare specific strategies. Future research should examine which implementation strategies are most effective in strengthening MPDSR fidelity, penetration, and sustainability in similar settings.

These findings have several implications for policy and practice across health system levels. At the facility level, strengthening adherence to MPDSR guidelines, such as fostering blame-free, confidential review environments, may be facilitated through ongoing training, mentorship, and supportive supervision for clinical and managerial staff. Facility leadership is able to play a critical role in promoting accountability and ensuring regular, well-attended review meetings, as well as tracking implementation of action plans. At the district and regional levels, investments in human resources, supervision systems, and monitoring mechanisms may be needed to support consistent implementation, including timely reviews and follow-up on recommendations. Nationally, policymakers may consider reinforcing MPDSR guidelines by allocating dedicated resources, integrating monitoring of implementation quality into routine health information systems, and supporting strategies that improve sustainability, such as standardizing tools for tracking recommendations and strengthening feedback loops. Together, these actions may help translate MPDSR processes into meaningful improvements in quality of care and reductions in maternal and perinatal mortality.

The implementation and sustainability of MPDSR in rural health facilities relies on several multilevel and multilayered factors. Through in-depth interviews with health facility staff in Kigoma Region, Tanzania, we identified important barriers and facilitators to MPDSR implementation outcomes. MPDSR provides an opportunity for health facilities and systems to understand causes of maternal death, address gaps in care, and prevent future maternal deaths.

## Supporting information

S1 Table Description of subthemes and illustrative quotes from qualitative, in-depth interviews with 17 delivery care providers (e.g., doctors, midwives, anesthesiologists) and 5 health administrators who oversaw or facilitated the facility’s MPDSR process, Kigoma Region, Tanzania. (DOCX)
