# Supplementary material for: Facility staff perspectives on the implementation of Maternal and Perinatal Death Surveillance and Response in six health facilities in Kigoma, Tanzania
Source: PLoS One. 2026 Jun 1;21(6):e0349233. doi: 10.1371/journal.pone.0349233 (PMC13225644; doi:10.1371/journal.pone.0349233)
Supplement: S1 Table — (DOCX) [file pone.0349233.s001.docx]

**S1 Table. Description of subthemes and illustrative quotes from qualitative, in-depth interviews with 17 delivery care providers (e.g., doctors, midwives, anesthesiologists) and 5 health administrators who oversaw or facilitated the facility’s MPDSR process, Kigoma Region, Tanzania.**

| **Major themes and Subthemes** | **Illustrative quotes** |
| --- | --- |
| Organizational and staff perspectives on the intervention | |
| **Organizational and staff perspectives on MPDSR’s benefits and impact** | *"There are a lot of good benefits of MPDSR. The health personnel who participate [in MPDSR] will know what was wrong and how to improve care. Also, we get updates about how to provide services and reduce maternal and perinatal death. Also, we get data which we can use to compare like last year and this year, by how many percent have death rate reduced, and also to know cause of death of last week and this week is what and to make intervention and improve well."*   - Nursing Officer and MPDSR Secretary     *"With action plans, when we follow up them and after we start implementing them, we are seeing that those knowledge gaps are phasing out and the death of that kind is also reduced. So, it's assisting with reducing maternal and perinatal death. And there are some SOPs, standard operating procedures, which have been formulated as a result of those review meetings."*   - Medical Doctor and MPDSR Focal Person     *"The biggest benefit of MPDSR is a platform to improve the knowledge of our own staff. Me, as a chairperson, I've learned a lot. I have learned a lot through attending MPDSR meetings. Not only professional knowledge, but there have several skills, the knowledge and administrative issues, how to overcome challenges. I mean, I've learned so many things through MPDSR meetings. I mean, it's very useful. It's a very simple question with so many answers."*   - Medical Doctor and MPDSR Chairperson |
| **Organizational and staff perspectives on MPDSR’s ease of use** | *“It happened once [that the assigned person failed to give feedback]. The only time it occurred we postpone the meeting, cause we did not think it was possible to discuss other action plans while were are not sure the previous action are completed.”*   - Assistant Nursing Officer and MPDSR Coordinator     *“In the action plan, the challenge is moving to the other facilities where the deceased was located/attended. There are costs, because we have to select members to go there as we need fuel so that they can go to the facilities involved. We also include the facility which referred the patient, because we need some information and some gaps which are found there. We want to know what problems there or if anything we can go and change there. We also include them in the meeting. They are invited guests, but not permanently, because some of the time we have all the information. Because at times you find the patient has just arrived from the referral facility and she collapses. So, you did not attend her, and you haven’t got any information, but it is a maternal death. So, we fail to discuss on the gaps because you just receive her and within half an hour she dies. Hence for us to conduct MPDSR review, we have to get information from there.”*   - Medical Doctor and MPDSR Chairperson     *“I think we have several issues in the general operationalization of MPDSR. For example, I know in the guideline it just tells you that all the people who were involved in treating a certain patient they need to attend the meeting. But suppose the one who treated the person is a hundred kilometers from here. Two hundred, four hundred kilometers from here. What does the guideline say? [Nothing.]…So, we have to support them to come. We have to find a mechanism of supporting them to come and attend the meeting so that they assist in our review. So yeah, we, I think we have several modifications we do just for the benefit of the meeting.”*   - Medical Doctor and MPDSR Chairperson and Focal Person |
| **Organizational and staff perspectives on organizational readiness to implement MPDSR** | *“The entire team in the review creates [action plans]. For example, when you are reviewing the case as the entire committee, the MPDSR focal person is the one who has the form and completes it, not by his own ideas but from all people’s ideas, i.e. here we fill so and so the gap and you all agree that that was the gap.”*   - Nursing Officer and District Coordinator     *“The readiness of the implementor and his companions, meaning the readiness to receive the target he wants to achieve enables the implementation of response. Also, resources as I had said... But we are trying our best.”*   - Medical Doctor and MPDSR Chairperson     *“It is commitment [that enables the implementation of response within the specific allocated time frame], because at times you find you have assigned someone to implement a certain task and we need feedback after a certain time, and it is something that he is really enthusiastic about, so he delivers it on time.”*   - Nursing Officer and MPDSR Secretary |
| Characteristics of the implementation setting | |
| **Characteristics of the implementation setting: limited human, financial, other resources** | *“It's a lack of resources here, lack of finances, lack of you know, it's always about lack of something, and it's not about the specific human entities because people are really devoting themselves. Actually, we have limited resources. It's a limited resource center, and it's a busy center… Some months are extremely busy, so, it's all about resources.”*   - Medical Doctor and MPDSR Chairperson     *“We have challenges [to implementing action plans] sometimes in human resources or fund resources… Another challenge is lacking of some equipment. For example, we can talk about lack of equipment from MSD. Sometimes you need a delivery kit, or you need maybe a baby warmer. It’s true that we have money, which was deposited by the government, but still when you order sometimes, we made a special order but you don’t get the things from MSD. Therefore, is the lack of equipment which might help a baby or mother. It depends on the environment.”*   - Nursing Officer and MPDSR Secretary     *“Provision of supplies is still a challenge. Till now, we have not provided supplies to reach our goals…I do not think [we have ever tried even once]. Not to my awareness. However, we usually provide them with transport if it is need during the implementation.”*   - Assistant Nursing Officer and MPDSR Coordinator     *“Monetary resources can affect the reviewing process a lit bit, such as when you are in review and stationary is needed and sometimes drinking water. Other members might tell you they don’t have transport fee because they live far. Things like those.”*   - Assistant Nursing Officer and MPDSR Coordinator |
| **Characteristics of the implementation setting: competing roles and responsibilities** | *“The greatest challenge is time resources, because I as the MPDSR secretary have to do my responsibilities in the ward in providing services and passing through members of MPDSR and encouraging them on the implementation of action plans, because they are people and at times they forget. So, I have to work on MPDSR and at the ward, but you are only one person. So, time resource is the greatest challenge.”*   - Assistant Nursing Officer and MPDSR Secretary     *“During implementation, the challenge is at times that you find you gave a task to someone and he/she doesn’t implement it fully 100% claiming to have overwhelming schedules. You know that there are different duties that may hinder him/her from completing the tasks.”*   - Medical Doctor and MPDSR Chairperson     *“There are many challenges [on following up the implementation of action plans], such as the responsible person failed to implement. For example, they were instructed to give health education but didn’t and they have a genuine reason. For example, due to shortage of staffs, perhaps is the moment which want them to be fulfilled but it is also the time when you are needed to prepare monthly report. Therefore, you might find someone is trying to catch up with time. For example, in the RCH clinic, the nurse might have 4 registries which need to be reported. Such as registry of maternal and baby report book. Chasing the deadline of preparing report at the same time, she is needed to implement a certain action plan, which led to a delay.”*   - Assistant Nursing Officer and MPDSR Secretary |
| Implementation and sustainability infrastructure | |
| **Implementation and Sustainability Infrastructure: training and mentorship** | *“My recommendation is to implement MPDSR trainings, because when the trainings were introduced, we went like 5 people from the district, the DMO, secretary, I, DNO, MOI and his matron from the hospital, matron from health center and another. So, we went like 7 people in the district and that means lots of people haven’t attended the training. It would have been better that even if it is not at the regional level but at the district level so that all staffs could understand what MPDSR is and how the reviews are conducted.”*   - Nursing Officer and Reproductive and Child Health Coordinator     *“The challenge that I had faced in extreme was in facility X which I had mostly been doing follow-up, I found that had a problem for example when you ask for a narrative summary staff start to fear because they think that perhaps someone is going to sue them. Then I reported to them to the senior, and the senior talked to them without success and forwarded it to the next senior. Then we decided that that person should come at the district level to get capacity building trainings.”*   - Nursing Officer and MPDSR Focal Person     *“It’s the training we received, but also the ones who received training thereafter came back to train us because not all of us received training. There is something called CME (continuous medical education). We still continue with it. But also on job trainings, when a case happens, you see how its solved and then we implement.”*   - Nursing Officer and Member of Emergency Response Team     *“The thing which I like to share with you is to request any special training, to train us because me as myself I am working out of experience and by passing through the guideline of MPDSR. Also, the focal person was not given training.”*   - Nursing Officer and MPDSR Secretary |
| **Implementation and Sustainability Infrastructure: facility leadership in organizing, chairing and supporting multidisciplinary meetings that follow rules of conduct** | *“The secretary informs the members about the meeting. And let's say tomorrow we have a MPDSR meeting. And then on the day of meeting the chairperson, MPDSR chairperson welcomes the members and make sure that every member has arrived on time. And before starting this session, he reminds the members that the meeting's purpose is to identify gaps. And confidentiality is observed. And there is no naming, no blaming is observed, and no shame so that everyone is free to express what he feels and what he knows.”*   - Medical Doctor and MPDSR Focal Person     *“Anonymity is very essential in our health cadres. There is patient confidentiality and as well as amongst us, not publishing information and so on… Before we start the review, we always share [the code of conduct]. That death has happened and that not blaming you are the one who caused it. We just look where were the gaps so that we can make improvements. So we always share the ethics before the MPDSR review.”*   - Medical Doctor and MPDSR Chairperson     *“Disciplinary actions are taken but we don’t use the MPDSR review meeting to make them, because we are afraid people might start to be afraid to come to the meeting because of the disciplinary actions. However, in the guidelines, there are instruction to take disciplinary action if it is found that the healthcare provided was a direct cause of death, but we don’t usually take them because of members might start to be scared to attend MPDSR meetings. But there are protocols which we do as a region when we see there a problem in place X, and it is related to the health care provider then we have special team in the region which comprises of the technical people. They will go and get information then after that they will bring feedback to RMO, after that RMO will give instruction  that to do A,B,C, and there are some people who were sent to disciplinary team but we don’t do it in the MPDSR meeting because people run from you and they will be afraid to talk, and we want people to be free. Unless the person is direct related to the maternal death.”*   - Nursing Officer and Maternal and Child Health Services Coordinator     *“Among the disciplinary actions we take, we have instruction letters and warning letters. But also in the past two years, there were some members who were given letters by the DMO (District medical officer) that they haven’t attended meetings several times. Even recently the DMO responded by sending letters to some of members who didn’t attend some meeting as some of meetings were sat during weekends. It was a bit challenging, and letters were written (to the addressee). Because it was still challenging, we saw it was not okay doing meetings on weekend, so we recently agreed that the meeting shouldn’t be held on weekends because the same person also has social activities.”*   - Nursing Officer and Member of the Emergency Response Team |
| **Implementation and Sustainability Infrastructure: data analysis and dissemination** | *“We use that data, and the most common one will be given priority in solving it. For example, if the problem was, let's say skills, so we put on a plan on giving staffs the skills and if the cause let's say was equipment, so we put it on the plan on solving it. But, we don’t prepare reports for it specifically. Maybe previously, because I was employed on 2022 and I have about only one and a half years.”*   - Medical Doctor and MPDSR Chairperson     *“We do analyze and create reports from the data collected from the reviews. We have something called the health governing committee of which its chairman is from the community and the secretary is the medical officer in charge. They are selected and usually they must be nine coming from NGO’s, different religious groups, people with influence and schools. So, through them, the information is analyzed. And they also sit quarterly and, in every quarter, we display the number of mortalities that occurred and then thereafter convey it to communities.”*   - Medical Doctor and MPDSR Chairperson     *“I haven’t ever dealt with analyzing or creating reports of the data collected from the reviews. But, of course, we do display the data through community gatherings like perhaps a politician or councilor is talking to the community or when a community maternal death has occurred, so one (health provider) is called to talk, he explains the cause of death, how big is the number of maternal community death, and how people should live.”*   - Medical Doctor and MPDSR Chairperson |
| **Implementation and Sustainability Infrastructure: designing quality improvement activities and annual health facility planning that incorporates MPDSR budgeting for implementation and follow up** | *“The good thing is that most of [recommendations] fall on the medical supplies and medical equipment. Another issue is on training, but it is there in the annual plan. So, we can have a budget for it. That's why we even have a budget for meetings. If it happens the things like we said that has more cost than we send that on the annual plan, so that you can have a budget on the next year. But if it's less cost, we can buy immediately.”*   - Medical Doctor and MPDSR Chairperson     *“We integrate recommendations into the annual health facility plans, but it depends. That's why I have said that it depends on the duration of the recommendation. You can have a gap, but to solve it needs maybe two to three years. For example, here at our facility we don't have a pediatric ward. We don't have it. And to implement, it is not simple. So, we always recommend it to the facility annual plan so that they can be included.”*   - Medical Doctor and MPDSR Chairperson and Focal Person     *“I am in the quality improvement team, and I have already given my recommendations on how to improve the services we provide. And today the quality improvement team was going to sit for a meeting. So, recommendations are delivered as the secretary is also part of the team. Currently, it is about demonstration of how to do some things (procedures) according to specific departments… and we do sit like four times a year, and we have sat a lot of meetings and train each other. It is a continuous process.”*   - Assistant Nursing Officer and MPDSR secretary. |
| **Implementation and Sustainability Infrastructure: district and regional support** | *“The region follows-up on action plans in many ways. They can do it by phone call or by supportive supervision and at times when a problem happens. The regional team then comes here.”*   - Nursing Officer and Reproductive and Child Health Coordinator     *“There is a mentor. There is a regional mentor. We are told perhaps his name is so and so and he will come for follow-up of action plans. But also, the district or the region usually come for follow-up and see is what we have written are the ones implemented…”*   - Nursing Officer and MPDSR Secretary     *“The district or region follows up on action plans through meetings, i.e.  we first have the facility MPDSR review that involves representatives from the district and then we have the district MPDSR review which must have the regional representative. That is where someone follows-up.”*   - Medical Doctor and MPDSR Chairperson |
| **Implementation and Sustainability Infrastructure: Community engagement** | *“For recommendations that require the community to get information, we usually use a radio, and unfortunately now are beginning to not have that to reach them so we use to communicate to them through radios. Most of the messages are to remind them the importance of attending or seeking care early before complications set in, because we are receiving patients in a very critical state. They are coming late from the community, and we think that is caused by knowledge gap that the community are not aware if I go late, they can save me even if I go late. And some of them, they even don't know the danger signs when to go to hospital. So we are giving this information.”*   - Medical Doctor and MPDSR Focal Person     *“Recommendations can be fed back to the community through radio or through meetings (conferences) or through community MPDSR. For example, a death occurred at the facility. The mother delivered at home. The baby died. While they found transport to another facility, the mother arrived dead. So, me, the district medical officer and the district nurse officer and the community health workers, district executive and village chairperson went there during the burial day. We were given a chance, and we provided education and they (men/fathers) left blaming each other that their wives hide lot of information from us like being in labor as they (wives) are afraid of being sent to the health facility earlier. And they understood us and from that day we did not have any maternal death.”*   - Nursing Officer and Reproductive and Child Health Coordinator     *“The facility does provide feedback about maternal and perinatal deaths to the community, because when they provide the education, they say they wish that no maternal or perinatal death should occur. That means the family, the community and the RCH and other staff should make sure that the mother should be taken care of, till she delivers and that shouldn’t be left to fathers only. But also, the community and the community leaders should know that in a certain place there is a pregnant woman and check to see if she attends clinic so that they can remind the fathers too.”*   - Nursing Officer and MPDSR Focal Person |
